# The repair Schwann cell and its function in regenerating nerves

**DOI:** 10.1113/JP270874

**Published:** 2016-03-21

**Authors:** K. R. Jessen, R. Mirsky

**Affiliations:** ^1^ Department of Cell and Developmental BiologyUniversity College LondonGower StreetLondonWC1E 6BTUK

## Abstract

Nerve injury triggers the conversion of myelin and non‐myelin (Remak) Schwann cells to a cell phenotype specialized to promote repair. Distal to damage, these repair Schwann cells provide the necessary signals and spatial cues for the survival of injured neurons, axonal regeneration and target reinnervation. The conversion to repair Schwann cells involves de‐differentiation together with alternative differentiation, or activation, a combination that is typical of cell type conversions often referred to as (direct or lineage) reprogramming. Thus, injury‐induced Schwann cell reprogramming involves down‐regulation of myelin genes combined with activation of a set of repair‐supportive features, including up‐regulation of trophic factors, elevation of cytokines as part of the innate immune response, myelin clearance by activation of myelin autophagy in Schwann cells and macrophage recruitment, and the formation of regeneration tracks, Bungner's bands, for directing axons to their targets. This repair programme is controlled transcriptionally by mechanisms involving the transcription factor c‐Jun, which is rapidly up‐regulated in Schwann cells after injury. In the absence of c‐Jun, damage results in the formation of a dysfunctional repair cell, neuronal death and failure of functional recovery. c‐Jun, although not required for Schwann cell development, is therefore central to the reprogramming of myelin and non‐myelin (Remak) Schwann cells to repair cells after injury. In future, the signalling that specifies this cell requires further analysis so that pharmacological tools that boost and maintain the repair Schwann cell phenotype can be developed.

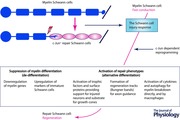

## Introduction

The striking regeneration potential of the peripheral nervous system is clearly illustrated by comparing the outcome of a blunt injury of the spinal cord (contusion or crush) with a similar injury to the sciatic nerve in rodents. Crushing the spinal cord is followed by the formation of a fluid‐ or matrix‐filled lesion, axonal retraction, retention of myelin debris distal to the injury, and absence of any significant axonal regeneration (reviewed in Beattie *et al*. 1997; Vargas & Barres, 2007; Plemel *et al*. 2008). After sciatic nerve crush, on the other hand, axons grow readily back to their targets, redundant myelin is removed and new myelin formed around regenerated axons, with the result that nerve tissue that is broadly normal in structure and function is restored in a surprisingly short time, 3–4 weeks (reviewed in Glenn & Talbot, 2013; Scheib & Höke, 2013; Brosius Lutz & Barres, 2014).

What is the cellular basis for the fundamental difference between these two tissues, similar though they are with respect to key components: axons, myelin and glial cells? In particular, what happens in the injured peripheral nerve to make repair so straightforward in this type of experiment?

Ultimately the answer relates to a general realization emerging from studies on cell differentiation, namely that the differentiated state of mammalian cells is less stable than previously thought. It is now clear that many cell types can be induced to change their differentiation state by experimental manipulation. Most often this has involved enforced expression of transcription factors in the culture dish to convert one cell into another (reviewed in Eberhard & Tosh, 2008; Yamanaka & Blau, 2010; Sisakhtnezhad & Matin, 2012). But there is also increasing evidence for comparable events *in vivo*, where the cell type conversions typically take place as helpful, or adaptive, responses to injury. A classic example is the conversion of pigment epithelium of the eye into lens epithelium, which helps restore the lens after eye injury in some newts and frogs (reviewed in Tsonis *et al*. 2004). This adaptive cellular reprogramming, induced by injury and promoting repair, can also be seen in mammalian tissues, including skin, liver, ear (reviewed in Jessen *et al*. 2015 *a*) and, as it turns out, peripheral nerve.

Damage to a peripheral nerve, whether crush or cut, triggers extensive changes in the differentiation state both of the injured neurons and of the Schwann cells distal to the injury. The neurons change expression of hundreds of genes, including a large number of transcription factors. This response (often referred to as the cell body reaction or signalling to growth mode switch) allows the neuron to shift its function from cell–cell signalling to that of building a new axon (Blesch *et al*. 2012; reviewed in Fu & Gordon, 1997; Doron‐Mandel *et al*. 2015). Equally strikingly, the myelin and non‐myelin (Remak) Schwann cells distal to nerve injury undergo a large scale change in gene expression, probably involving some thousands of genes, and change function from maintenance of axonal ensheathment and myelin to that of supporting regeneration (Nagarajan *et al*. 2002; Bosse *et al*. 2006; Barrette *et al*. 2010; Arthur‐Farraj *et al*. 2012). Because these cells are specialized for repair and differ from other cells in the Schwann cell lineage, as described below, we refer to these cells as repair Schwann cells (or Bungner cells since they form guidance tracks for regenerating axons called Bungner's bands) (Arthur‐Farraj *et al*. 2012; reviewed in Jessen *et al*. 2015 *b*).

In sum, peripheral nerves owe their regenerative potential to the flexible differentiation state of PNS neurons and Schwann cells, and their ability to convert to cells devoted to repair after injury. In the CNS, this adaptive injury response is generally subdued in neurons (reviewed in Bradke *et al*. 2012; Doron‐Mandel *et al*. 2015), and oligodendrocytes, the myelin forming cells of the CNS, are not reprogrammed to repair cells and serve no helpful function for regeneration distal to injury (reviewed in Vargas & Barres, 2007).

**Figure 1:**
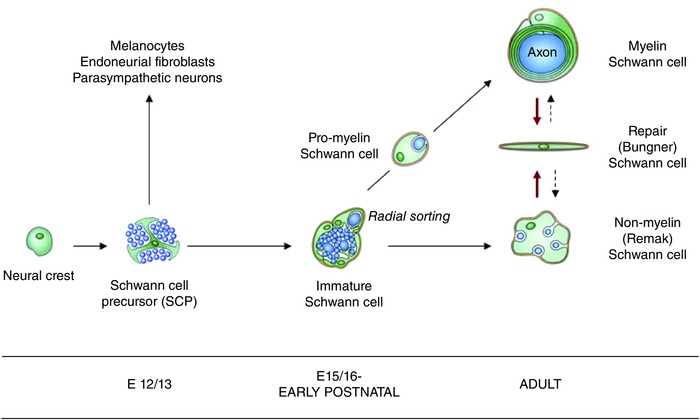
**The repair (Bungner) Schwann cell in a developmental context** The diagram shows the repair (Bungner) Schwann cell, and the key stages of Schwann cell development, in addition to other developmental options for the Schwann cell precursor (Jessen & Mirsky, 2005). Arrows indicate developmental and injury‐related transitions. Black continuous arrows: normal development. Red arrows: the Schwann cell injury response. Dashed arrows: post‐repair re‐formation of myelin and Remak cells. Embryonic dates (E) refer to mouse development (from Jessen *et al*. 2015 *b*).

**Figure 2:**
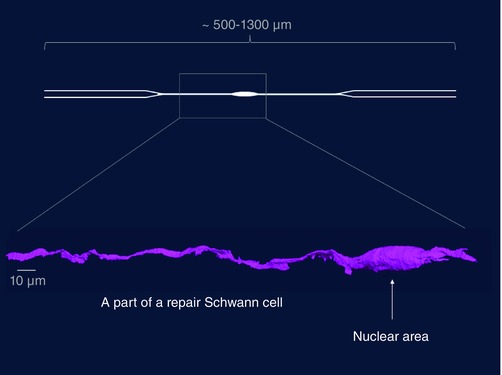
**The structure of repair Schwann cells** A repair Schwann cell in the distal stump (4‐week transected tibial nerve without re‐innervation), as shown by serial block face scanning electron microscopy. Only a part of the cell is shown, as indicated by the box superimposed on a schematic diagram of a repair cell (R. Mirsky, K. R. Jessen H. Armer and P. Munro, unpublished).

This article will focus on the repair (Bungner) Schwann cell in nerves distal to crush or cut injury (Figs 1 and 2). We will describe the properties, generation and maintenance of this cell, and its recently discovered function in myelinophagy, the autophagic breakdown of redundant myelin in injured nerves.

## The Schwann cell injury response: de‐differentiation, activation or reprogramming

Although it is obvious that the axonal death distal to nerve crush (or cut) is accompanied by a radical phenotypic change in Schwann cells, there has long been disagreement in the literature about how this change is best understood. Some groups view the Schwann cell injury response as de‐differentiation (e.g. Chen *et al*. 2007; discussed in Jessen & Mirsky, 2008), while others see it as activation (Armstrong *et al*. 2007; Campana, 2007; Webber & Zochodne, 2010; Allodi *et al*. 2012). The dual nomenclature is confusing, because these terms appear to contradict each other by implying loss or gain of phenotypes respectively. But it is likely to have a simple explanation, which is that that the Schwann cell injury response in fact involves both of these processes. Thus after injury, myelin Schwann cells lose their characteristic gene expression pattern, which represents loss of differentiated features, de‐differentiation, but simultaneously they activate a set of repair‐related phenotypes, a repair programme, as detailed in the following section.

Previously we pointed out that the generation of repair Schwann cells could be compared to injury responses in other tissues, where cells also change phenotype to promote healing (adaptive reprogramming) (Jessen *et al*. 2015 *a*). Notably, these cell type conversions also involve the combination of de‐differentiation and activation. Thus, the injury‐induced conversion of pigment‐to‐lens epithelium in the newt involves the depigmentation and inhibition of melanogenesis (de‐differentiaton), but also the gain (activation) of crystallin synthesis to form the transparent lens (reviewed in Shen *et al*. 2004; Tsonis *et al*. 2004). Similarly in mammals, the conversion of α‐cells to β‐cells in the pancreatic islets following destruction of β‐cells involves loss of glucagon expression (de‐differentiation) and gain (activation) of insulin expression (Thorel *et al*. 2010; Chera *et al*. 2014). Such cell type conversions are generally referred to as transdifferentiation or direct (or lineage) reprogramming (Graf & Enver, 2009; Sisakhtnezhad & Matin, 2012). These concepts and terminology appear equally applicable to the Schwann cell injury response, reflecting a more accurate understanding than de‐differentiation or activation, words that refer only to a part of the underlying process (reviewed in Jessen *et al*. 2015 *a*).

## Key events in Schwann cell reprogramming

Axons can be interrupted in two ways, by nerve cut or crush. After crush, the basal lamina tubes around individual axon/Schwann cell units are intact, and an axon remains within its native basal lamina sheath as it regenerates into the distal stump. After cut, the connective tissue and basal lamina sheaths are interrupted. If cut nerves are not repaired by re‐attaching the proximal and distal stumps, a tissue bridge forms between the two ends of the nerve, through which axons accompanied by Schwann cells, regeneration units, grow towards the distal stump guided by fibroblasts and blood vessels, meeting Schwann cell outgrowth from the cut end of the distal stump (Morris *et al*. 1972; Friede & Bischhausen, 1980; Meller, 1987; Barrette *et al*. 2008; Parrinello *et al*. 2010; Cattin *et al*. 2015). The Schwann cells of the regeneration units that project from the proximal stump are the daughter cells of the cells associated with cut axons just proximal to the injury. Although they have abandoned myelin differentiation, they may never have lost contact with axons, and the exact differentiation state of these cells is not clear. Nerve repair by re‐attaching the cut ends, a standard clinical treatment, leaves only a microscopic gap to be filled by a bridge and regeneration units.

Irrespective of whether axons are severed by crush or cut, the Schwann cell injury response in the nerve stump distal to the injury is similar. For simplicity this response will be discussed here in terms of myelin Schwann cells only; similar principles are likely to hold for non‐myelin (Remak) cells. The injury response can be viewed as having two principal components.

One of these is the reversal of myelin differentiation. Genes coding for the key myelin transcription factor Egr2 (Krox20), the enzymes of cholesterol synthesis, structural proteins such as P0, myelin basic protein (MBP), and membrane associated proteins like myelin associated glycoprotein (MAG) and periaxin are all rapidly down‐regulated (reviewed in Chen *et al*. 2007; Jessen & Mirsky, 2008). Conversely molecules that characterize pre‐myelinating Schwann cells in developing nerves (immature Schwann cells), including L1, neural cell adhesion molecule (NCAM), p75 neurotrophin receptor (p75NTR) and glial fibrillary acidic protein (GFAP), are up‐regulated (Chen *et al*. 2007; Jessen & Mirsky, 2008).

The second and vital constituent of the injury response involves the novel appearance of a set of phenotypes which are not active in Schwann cells in normal mature nerves or in Schwann cells in developing nerves. This repair programme includes a number of components. First, the up‐regulation of neurotrophic factors and surface proteins that promote axonal elongation and the survival of injured neurons, including glial cell line‐derived neurotrophic factor (GDNF), artemin, brain‐derived neurotrophic factor (BDNF), neurotrophin‐3 (NT3), nerve growth factor (NGF), vascular endothelial growth factor (VEGF), erythropoietin, pleiotrophin, p75NTR and N‐cadherin (Fontana *et al*. 2012; Brushart *et al*. 2013; reviewed in Boyd & Gordon, 2003; Chen *et al*. 2007; Scheib & Höke, 2013; Wood & Mackinnon, 2015). Second, it involves the activation of an innate immune response, including the upregulation of cytokines including tumour necrosis factor α (TNFα), interleukin‐1α (Il‐1α), Il‐1β, leukaemia inhibitory factor (LIF) and monocyte chemotactic protein 1 (MCP‐1) by the Schwann cells in the distal stump (reviewed in Martini *et al*. 2008; Rotshenker, 2011). This allows repair Schwann cells to interact with immune cells and, in particular, recruit macrophages to the nerve. This immune response likely promotes nerve regeneration in numerous ways. Cytokines such as Il‐6 and LIF not only attract macrophages to the injured nerve but can also act on neurons to promote axonal regeneration (Hirota *et al*. 1996; Cafferty *et al*. 2001; reviewed in Bauer *et al*. 2007). In addition, macrophages that invade nerves and ganglia provide an additional sustained source of cytokines, promote vascularisation of the distal nerve (Barrette *et al*. 2008; Niemi *et al*. 2013; Cattin *et al*. 2015), and co‐operate with Schwann cells to degrade myelin debris that potentially inhibits axon growth during the second phase of myelin clearance (see further below) (reviewed in Hirata & Kawabuchi, 2002; Rotshenker, 2011). Third, injury prompts the formation of regeneration tracks to help guide the growth of axons. The repair Schwann cells adopt an elongated bipolar morphology (Fig. 2) and align in columns (bands of Bungner) inside the basal lamina tubes that previously enclosed either myelin or Remak cells and their associated axons prior to injury (Stoll & Müller, 1999). This structure provides essential substrate and guidance cues to enable regenerating axons to reconnect with their target tissues. The fourth component of the repair programme is the activation of autophagy for myelin breakdown, which will be discussed in the following section.

Together the activation of the de‐differentiation and repair programmes recasts Schwann cells of intact nerves as cells that are equipped in a number of ways to promote regeneration, namely as repair (Bungner) Schwann cells. These cells ensheath axons and transform back to myelin and Remak cells in regenerated nerves. The repair Schwann cell is therefore a transient cell state that meets the particular demands that arise in injured tissue.

## Schwann cells clear myelin by activation of myelinophagy

Surprisingly, during the first 5–7 days after injury Schwann cells themselves take a major part in breaking down their own redundant myelin sheaths. It has been estimated that about 50% of the myelin is degraded during this first phase of myelin clearance (Perry *et al*. 1995; see also Niemi *et al*. 2013). The second phase of myelin clearance is dominated by macrophages, which gradually accumulate in damaged nerves and phagocytose myelin debris (Ramaglia *et al*. 2008; Vargas *et al*. 2010; reviewed in Hirata & Kawabuchi, 2002; Dubový *et al*. 2013). During injury‐induced Schwann cell reprogramming, therefore, myelin cells not only switch off myelin maintenance, but also switch on an intracellular pathway for myelin destruction. Myelin breakdown by Schwann cells is often described as phagocytosis (e.g. Stoll & Müller, 1999; Hirata & Kawabuchi, 2002), although earlier authors were more cautious (Holtzman & Novikoff, 1965). But this notion is problematic because phagocytosis is primarily a mechanism for ingestion of extracellular material, while myelin is from the start a Schwann cell (membrane) component, and there is no evidence that myelin separates from Schwann cells to a significant extent during the first, Schwann cell‐dependent, phase of myelin clearance.

**Figure 3:**
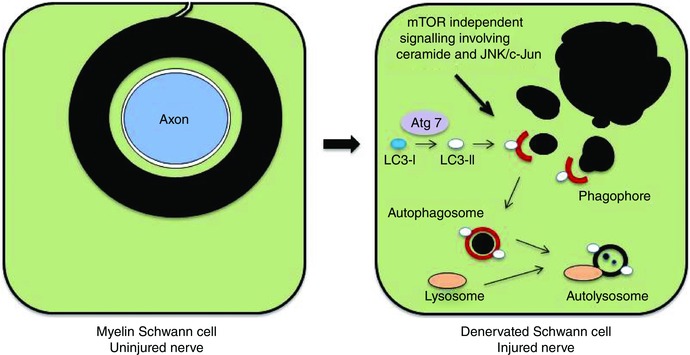
**Outline of myelinophagy** Left diagram: a transverse section through a myelin Schwann cell in an uninjured nerve. The myelin sheath is in direct continuity with the Schwann cell membrane and a component of the Schwann cell. Right diagram: a myelin Schwann cell after nerve injury and axonal degeneration. The myelin sheath has broken up into myelin ovoid and smaller fragments lying in the Schwann cell cytoplasm. The proposed role of autophagy in digesting these fragments is illustrated (from Gomez‐Sanchez *et al*. 2015).

This problem has been revisited in recent work. This shows that the first step in myelin breakdown is the activation of an actin‐dependent process for dividing the myelin sheath into discrete oval‐shaped intracellular segments, which gradually break up into smaller myelin remains (Jung *et al*. 2011). Our own work indicates that these myelin fragments are delivered to lysosomes for digestion by a form of selective, mechanistic target of rapamycin (mTOR)‐independent autophagy, myelinophagy, which is distinct from the classical, mTOR‐dependent starvation autophagy mechanisms (Gomez‐Sanchez *et al*. 2015). Autophagy is strongly activated in injured Schwann cells, and myelin debris can be directly seen in double membrane autophagosomes, the distinctive intracellular ferries that deliver cargo to lysosomes during the autophagy process. Pharmacological or genetic autophagy inhibition also inhibits myelin breakdown (Fig. 3). Autophagy of myelin has also been seen in Schwann cells during axonal degeneration in the dental pulp (Suzuki *et al*. 2015).

The identification of autophagy as the key process by which Schwann cells do away with their myelin sheaths as they convert to repair cells makes biological sense, because autophagy is the typical mechanism by which cells digest their own components, organelles and large macromolecular complexes. Schwann cell phagocytosis of extracellular myelin debris may well play a part during the second macrophage‐dominated phase of myelin clearance.

It would appear to be potentially hazardous for the Schwann cell to possess a pathway that can effectively destroy its own myelin sheath. It remains to be seen whether this self‐eating of myelin is liable to be inappropriately activated, and whether this plays a part in any of the large number of myelin disorders. The possibility that this may be the case is raised by our observation that in a mouse model of CMT1A, the most common hereditary demyelinating neuropathy in man, uninjured nerves show evidence of enhanced autophagy activation (Gomez‐Sanchez *et al*. 2015). Classical, mTOR‐dependent starvation autophagy can also be activated in Schwann cells where it can serve the beneficial function of degrading protein aggregates that form in some myelin mutants (Rangaraju *et al*. 2010).

It is intriguing that in the CNS the transected optic nerve, where myelin is not degraded after injury, shows little evidence of the activation of autophagy (Gomez‐Sanchez *et al*. 2015).

## A comparison of repair Schwann cells and immature Schwann cells

Myelin and Remak Schwann cells develop from immature Schwann cells in perinatal nerves (reviewed in Jessen & Mirsky, 2005). The de‐differentiation model of the Schwann cell injury response therefore predicts the generation of a cell in injured nerves that is similar to the immature Schwann cell found in developing nerves prior to myelination. Comparison of these two cells reveals, however, substantial differences.

First, they show a distinct molecular expression profile. This is exemplified by GDNF, oligodendrocyte transcription factor 1 (Olig 1), sonic hedgehog (Shh) and artemin, all of which are controlled by c‐Jun and highly up‐regulated in Schwann cells of injured nerves but absent/low in immature cells (Arthur‐Farraj *et al*. 2012; Fontana *et al*. 2012). These genes therefore serve as markers that distinguish repair Schwann cells in adult nerves from immature Schwann cells in perinatal nerves. One of these, GDNF, is detected in Schwann cell precursors of early embryonic nerves but is down‐regulated before birth (Piirsoo *et al*. 2010). Other genes including Olig1, Shh and artemin are absent/low both in immature cells and in earlier stages of the Schwann cell lineage (Lu *et al*. 2000; Zhou *et al*. 2000; Lin *et al*. 2015). Because these genes therefore appear to be expressed *de novo* after injury, they serve as distinctive markers of repair Schwann cells.

Further extensive differences in gene expression between immature and repair Schwann cells are indicated in a study comparing developing and regenerating nerves (Bosse *et al*. 2006). This work identified over a hundred genes that were regulated distal to nerve crush, although these genes were not developmentally regulated, being expressed at similar levels in newborn and adult uninjured nerves. Most of these genes were up‐regulated after injury, suggesting that induction of a large number of injury‐specific genes is one of the features that differentiate repair Schwann cells from cells in developing nerves prior to myelination.

Second, denervated cells, unlike immature cells, are engaged in a local, innate immune response, involving expression of a number of cytokines, and the attraction and activation of macrophages (reviewed in Martini *et al*. 2008; Gaudet *et al*. 2011; Rotshenker, 2011).

Third, an important function of denervated cells, but not immature cells, is to organize the clearance of myelin, indirectly by macrophage recruitment and directly by myelin autophagy (myelinophagy) (reviewed in Hirata & Kawabuchi, 2002; Gomez‐Sanchez *et al*. 2015; Suzuki *et al*. 2015).

Fourth, another key function of denervated cells is to guide growing axons to their target areas, which they accomplish by forming the Bungner regeneration tracks (Stoll & Müller, 1999). Perhaps surprisingly, a comparable guidance function is not shared by developing Schwann cells, because in embryos, peripheral axons can find their way to their target fields essentially normally in the absence of glial cells (Schwann cell precursors and immature Schwann cells) (Grim *et al*. 1992; Riethmacher *et al*. 1997).

Lastly, transcriptional controls differ in these two cell types, since c‐Jun is essential for the specification of the denervated cell, but this transcription factor appears to be dispensable for the generation of other cells in the Schwann cell lineage including functional immature Schwann cells (Arthur‐Farraj *et al*. 2012).

This comparison reveals two distinct Schwann cell phenotypes with separate functions and transcriptional controls. The essential role of immature Schwann cells is to act as a pool from which myelin and non‐myelin (Remak) cells develop, while Schwann cells in damaged nerves carry out specific functions related to regeneration and wound repair. This is consistent with the idea that the Schwann cell injury response represents a reprogramming process, which converts myelin and Remak Schwann cells to a Schwann cell specialized for repair (reviewed in Jessen *et al*. 2015 *a*,*b*).

## The generation of the repair Schwann cell: the role of c‐Jun

The transcription factor c‐Jun is low or absent in Schwann cell precursors, up‐regulated in immature Schwann cells but suppressed during postnatal development, although it remains detectable in many non‐myelin (Remak) cells, and to a lesser extent in myelin cells, in adult nerves (Parkinson *et al*. 2004; 2008; Arthur‐Farraj *et al*. 2012; Hantke *et al*. 2014; Klein *et al*. 2014). It has long been known that c‐Jun is rapidly induced to high levels in the Schwann cells of injured nerves (De Felipe & Hunt, 1994; Shy *et al*. 1996). The functional significance of this was demonstrated with the generation of mice in which c‐Jun is selectively inactivated in Schwann cells (c‐Jun cKO mice). Uninjured nerves are essentially normal in these mutants, indicating that c‐Jun is not essential for Schwann cell development. Axonal regeneration and functional recovery after injury are, however, strikingly compromised or absent. The regeneration failure in c‐Jun cKO mice is due to the central function of c‐Jun in Schwann cell reprogramming, since this factor controls both components of the Schwann cell injury response, de‐differentiation of myelin cells and activation of the repair programme (Arthur‐Farraj *et al*. 2012).

c‐Jun promotes de‐differentiation, being required for the normal suppression of myelin genes after injury, including the *Pzero* and *myelin basic protein* genes and the gene encoding the pro‐myelin transcription factor *Egr2* (*Krox20*). This suppressive function of c‐Jun and its cross‐antagonistic relationship with Egr2 (Krox20) was uncovered before its role in regeneration and helped give rise to the notion that c‐Jun, together with a number of other transcriptional regulators, such as Notch, Sox2, Pax3 and Id2, acted as a negative regulator of myelination (Parkinson *et al*. 2004; 2008; reviewed in Jessen & Mirsky, 2008). While many of these genes may be important for regulating the rate or onset of myelination during development, the key *in vivo* role for c‐Jun‐mediated suppression of myelin genes appears to be that of helping to supress myelin gene expression after injury.

c‐Jun is also essential for the normal activation of the repair programme, as seen from the following observations (Arthur‐Farraj *et al*. 2012; Fontana *et al*. 2012). First, in c‐Jun cKO mice the Schwann cells distal to injury fail to normally up‐regulate important trophic factors and cell surface proteins that support survival and axon growth, including GDNF, artemin and BDNF, p75NTR and N‐cadherin. Of these, GDNF and artemin have been shown to be direct targets of c‐Jun. Substantial numbers of dorsal root ganglion (DRG) sensory neurons and facial motoneurons die after sciatic and facial nerve injury, respectively, in c‐Jun cKO mice, revealing a key function for repair Schwann cells, and c‐Jun signalling, in support of neuronal survival. Second, because c‐Jun promotes myelinophagy, c‐Jun cKO nerves show long term delay in myelin clearance. Third, the regeneration tracks (Bungner bands) that denervated Schwann cells attempt to form without c‐Jun are structurally disorganized. In culture, c‐Jun in necessary for what has become known as the ‘typical’ narrow, bi/tripolar Schwann cell morphology, with c‐Jun‐negative cells tending to be flattened and sheet‐forming. Similarly *in vivo*, c‐Jun appears to be required for the conversion of the more complex and flattened structure of the myelin Schwann cell to the narrow and rod‐like morphology of repair cells which is required for the formation of normal regeneration columns.

Evidence is emerging that epigenetic mechanisms such as histone methylation state and miRNA also take part in the activation of the repair programme, since demethylation of H3K27 and down‐regulation of key miRNAs have been implicated in the activation of important injury factors including Shh, insulin‐like growth factor binding protein 2 (Igfbp2), Olig1 and GDNF (Lin *et al*. 2015; Ma *et al*. 2015).

The innate Schwann cell immune response to injury is to some extent regulated by c‐Jun because in cut nerves of c‐Jun cKO mice macrophage invasion is reduced at the injury site, and degenerating nerves in these mice contain large numbers of bloated macrophages. Many cytokines are, however, normally up‐regulated in the mutants and macrophage numbers are not significantly altered in crushed nerves or in cut nerves away from the location of the injury (Arthur‐Farraj *et al*. 2012). This suggests the participation of other pathways in driving the immune response. The extracellular signal‐regulated protein kinases 1 and 2 (ERK1/2)–mitogen‐activated protein kinase (MAPK) signalling pathway is activated in injured nerves and has been implicated in the control of immune functions in Schwann cells, in particular the activation of MCP‐1 expression, a key factor in attracting monocytes/macrophages to damaged nerves, and additional aspects of Schwann cell de‐differentation (Sheu *et al*. 2000; Harrisingh *et al*. 2004; Fischer *et al*. 2008; Groh *et al*. 2010; Shin *et al*. 2013). The other main MAPK signalling pathways, p38 and JNK, are also activated by nerve injury, and all three MAPK pathways have been implicated in promoting de‐differentiation of myelin cells (Myers *et al*. 2003; Parkinson *et al*. 2004; Yang *et al*. 2012). Demyelination after injury is also accelerated by Notch signalling, which is activated in Schwann cells in distal stumps. Inappropriate activation of Notch or Raf/ERK in Schwann cells of uninjured nerves is sufficient to cause demyelination, even in uninjured nerves (Woodhoo *et al*. 2009; Napoli *et al*. 2012).

## c‐Jun and nerve disease

As predicted from animal studies, in human nerves c‐Jun is also expressed at low levels in normal Schwann cells, but up‐regulated in a number of different neuropathic conditions that do not involve nerve cut or crush (Hutton *et al*. 2011). In line with this, c‐Jun is elevated in uninjured nerves of a mouse model of the most common human genetic demyelinating neuropathy, CMT1A (Hantke *et al*. 2014). If this is prevented by genetically inactivating c‐Jun, the CMT1A mice exhibit distal sensory axonopathy and deterioration in sensory–motor performance. In a mouse model of another human genetic demyelinating disease, CMT1X, c‐Jun levels are also increased in Schwann cells of uninjured nerves and at least one c‐Jun target, GDNF, is also elevated (Klein *et al*. 2014). In both of these mouse models, the c‐Jun elevation is seen in the nuclei of Schwann cells that retain myelin differentiation. Further, in a mouse mutant involving inactivation of the liver kinase B1 (LKB1) in Schwann cells and showing axonal damage without overt demyelination, c‐Jun protein is elevated as well as c‐Jun‐associated injury molecules including GDNF and Shh (Beirowski *et al*. 2014).

Taken together these observations raise two points. First, they indicate that significant c‐Jun elevation is compatible with myelin differentiation and does not cause demyelination. This is supported by our observations that a mouse engineered to show 5‐ to 8‐fold overexpression of c‐Jun protein in Schwann cells, nevertheless has relatively normal myelin sheaths. Although significant, these c‐Jun levels are about an order of magnitude lower than those seen after nerve cut (J. Gomez‐Sanchez, R. Mirsky and K. R. Jessen, unpublished). Second, they suggest that even in uninjured nerves, Schwann cells respond to adverse conditions, in these cases caused genetically, by relatively modest c‐Jun activation, which is low enough to be compatible with the myelin differentiation but high enough to activate repair‐related genes, representing a graded neuroprotective Schwann cell response to nerve distress that does not involve the overt generation of the repair phenotype associated with nerve injury.

## Maintenance of the repair phenotype

In line with the wide‐ranging role of c‐Jun in the specification of repair Schwann cells, reduced activation of Schwann cell c‐Jun in damaged nerves has recently been implicated in the age‐dependent reduction in regeneration that is typical of older animals (Painter *et al*. 2014). Another case of reduced c‐Jun expression is seen in Schwann cells in chronically denervated distal nerve stumps. In distal stumps of cut mouse nerves (without regeneration), c‐Jun levels are significantly lower at 10 weeks after injury than they are after 1 or 4 weeks (L. Wagstaff, K. R. Jessen and R. Mirsky, unpublished). This observation suggests that the repair phenotype is not stable, but fades with time. This is likely to be relevant in regenerating nerves of humans and other larger animals where the more distal Schwann cells are without axonal contact for many months while axons slowly elongate through the nerve. It is well established that during this time the axon‐free distal nerve stump gradually loses its repair‐supportive capacity, and that this decline is one of the key reasons for regeneration failure in humans (Höke, 2006; Sulaiman & Gordon, 2009). Therefore it will be relevant to determine whether reduced growth support is caused by reduced c‐Jun expression, and whether the Bungner repair phenotype can be maintained for long periods of time, or re‐activated, by promoting c‐Jun signalling. Analysis of the cell‐extrinsic and cell‐intrinsic pathways that maintain the repair phenotype, and the identification of pharmacological tools that promote this cell state (Heinen *et al*. 2015), is clearly an important future research direction.

## Additional information

### Competing interests

None declared.

### Author contributions

Both authors have approved the final version of the manuscript and agree to be accountable for all aspects of the work. All persons designated as authors qualify for authorship, and all those who qualify for authorship are listed.

### Funding

The work from the authors' own laboratory and quoted in this article was funded by Wellcome Trust Programme grants (091119 and 074665), an MRC project grant (G0600967) and grant agreement No. HEALTH‐F2‐2008‐201535 from the European Community's Seventh Framework Program (FP7/2007‐2013).
